# Development and Validation of a Neurosurgical Phantom for Simulating External Ventricular Drain Placement

**DOI:** 10.1007/s10916-024-02133-4

**Published:** 2025-01-03

**Authors:** Jesse A. M. van Doormaal, Tim Fick, Ernest Boskovic, Eelco W. Hoving, Pierre A. J. T. Robe, Tristan P. C. van Doormaal

**Affiliations:** 1 Department of Neurosurgery, University Medical Centre Utrecht, Utrecht, The Netherlands; 2 Department of Medical Technology and Clinical Physics, University Medical Centre Utrecht, Utrecht, The Netherlands; 3 Department of Neuro-Oncology, Princess Máxima Centre for Pediatric Oncology, Utrecht, The Netherlands; 4 Department of Neurosurgery and Klinisches Neurozentrum, Universitätsspital Zürich, Zurich, Switzerland

**Keywords:** External Ventricular Drain, Ventriculostomy, Surgical Training, Surgical Simulation, Surgical Phantom

## Abstract

This study aimed to develop and validate a cost-effective, customizable patient-specific phantom for simulating external ventricular drain placement, combining image segmentation, 3-D printing and molding techniques. Two variations of the phantom were created based on patient MRI data, integrating a realistic skin layer with anatomical landmarks, a 3-D printed skull, an agarose polysaccharide gel brain, and a ventricular cavity. To validate the phantom, 15 neurosurgeons, residents, and physician assistants performed 30 EVD placements. The effectiveness of the phantom as a training tool was assessed through a standardized user experience questionnaire, which evaluated the physical attributes, realism, and overall satisfaction. The mechanical properties of the phantom brain were quantified by measuring catheter insertion forces using a linear force tester to compare them to those experienced in real brain tissue. The study participants successfully completed EVD placements with a 76.7% optimal placement rate, which aligns with rates observed in clinical practice. Feedback highlighted the anatomical accuracy of the phantom and its value in enhancing surgical skills, though it also identified areas for improvement, particularly in the realism of the skin layer. Mechanical testing demonstrated that the insertion forces required were comparable to those encountered in actual brain tissue. The developed phantom offers a realistic, low-cost, and adaptable model for EVD simulation. This tool is particularly beneficial for both training and research, with future enhancements planned to improve the realism of the skin and incorporate more anatomical features to increase the fidelity of the simulation.

**Supplementary Information:**

The online version contains supplementary material available at 10.1007/s10916-024-02133-4.

## Introduction

Placement of an external ventricular drain (EVD) is a potentially lifesaving procedure generally performed in urgent brain conditions such as obstructive hydrocephalus (secondary to intraventricular/subarachnoid hemorrhage, meningitis, brain tumors or ventriculoperitoneal shunt failure) or traumatic brain injury[1, 2]. EVD placement is often taught to neurosurgical residents in an early phase of their training due to the relative simplicity and high prevalence of the procedure[3, 4]. Effective training of neurosurgical residents is mandatory for reducing complications and optimizing procedural time[5].

Training in neurosurgery is traditionally founded on apprenticeship under the guidance of experienced surgeons[6]. While this has been the golden standard for centuries, it presents certain limitations. Training on live patients, especially involving residents who lack significant surgical experience, may result in suboptimal care[7]. Furthermore, variable exposure to the procedure due to the unpredictable nature of clinical practice may impede standardization of neurosurgical training[8]. Cadaver-based training helps address issues regarding ethics and exposure, yet its availability is constrained by challenges in logistics and associated expenses[9]. Beyond EVD placement, scientific research aimed at developing surgical innovations faces comparable challenges, as testing new techniques on real patients raises ethical concerns[10], and the limited availability of cadavers restricts experimental opportunities.

Surgical simulation using anatomical phantoms provides a cost-effective alternative that circumvents such challenges. Simulation-based training can be beneficial to increase knowledge, technical skill and operating speed for surgical trainees [11–13]. Furthermore, surgical phantoms provide possibilities for scientific research and development of surgical innovations, as this often requires large volumes of procedures conducted within a controlled environment.

Advances in 3-D printing technology and imaging segmentation provide new options for creating relatively low-cost phantoms with biomimicking properties. Image segmentation facilitates usage of real patient data to create phantom components, while 3-D printing and molding techniques provide low-cost and rapid manufacturing of biomimicking anatomical structures. Using such techniques, previous studies developed phantoms for the simulation of EVD placement [14–17]. Other phantoms have been developed for skull base surgery [15] and third ventriculostomy [18–21]. Mechanical testing of materials used for manufacturing brain phantoms have indicated that specific concentrations of gelling agents such as agarose polysaccharide, gellan gum and polyvinyl alcohol display mechanical properties similar to mammalian brain tissue [22, 23]. Neurosurgeons and neurosurgical residents considered such phantoms adequately realistic to be used for technical skill training [14, 15, 18–21]. Despite promising results, many described phantoms require substantial time investment and specialized technical expertise. Furthermore, the number of phantoms dedicated to the simulation of EVD placement is limited and lack a realistic skin layer on the facial and forehead areas, which limits the realistic simulation of soft-tissue facial landmarks.

In this article, we present a semi-realistic phantom designed to simulate EVD placement, which is manufactured through a combination of image segmentation, 3-D printing and molding techniques. This combined approach aimed to create a phantom that is inexpensive, time-efficient and easily adaptable to individual patient imaging. The suitability of the phantom for surgical training was assessed using a multimodal approach. Usability was evaluated by testing the simulation with neurosurgeons and neurosurgical residents, and by administering user questionnaires. The mechanical properties of the phantom brain were analyzed by compiling the catheter insertion force profile and comparing it to that of real brain tissue using an experimental set-up. The radiological properties were analyzed by measuring Hounsfield Units using Computed Tomography (CT).

## Methods

### Segmentation

For the purposes of this proof-of-principle study, 2 phantom variations, each representing varying levels of technical complexity associated with EVD placement, were designed and fabricated. Two contrast-enhanced T1 Magnetic Resonance Imaging (MRI) scans were selected from an anonymous secured database previously collected by the University Medical Centre Utrecht's clinical trial bureau [24, 25]. To create challenging phantoms, patients with bicaudate indices of 0.1335 and 0.1201 were selected, with corresponding Evans indices of 0.2333 and 0.2645. Corresponding contrast-enhanced T1 Magnetic Resonance Imaging (MRI) scans of these cases were segmented and converted to 3-D models of the skin, brain and ventricles using a neurosurgical software package for automatic image segmentation (Lumi, Augmedit, Naarden, The Netherlands).

### Outer Shell Manufacturing

**Fig. 1:**
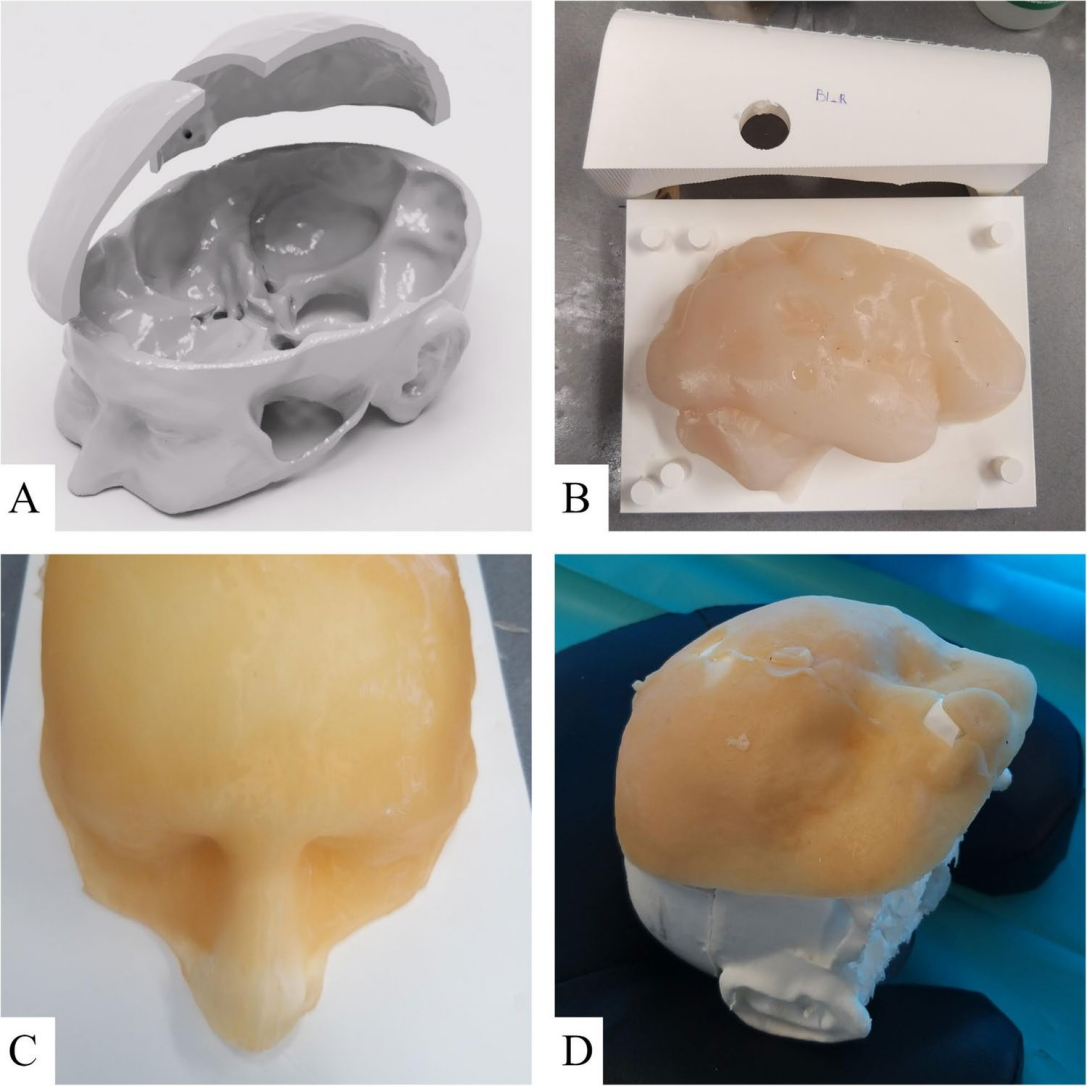
A 3-D printed modular skull. B Molds with hemisphere casted in 1.0% agarose solution. C Molds for casting skin with gelatin/glycerin skin layer. D Finished phantom

Using the skin segmentation and a standardized 3-D model of an adult human skull, we created an outer shell for the phantoms. This shell consisted of the skull with segmented anatomical landmarks in the facial (medial canthus, lateral canthus, nasion) and auricular region (tragus, helix, and antihelix). The skull also featured a coronary, sagittal and occipital suture. The shell was 3-D printed in polylactic acid (PLA) using a layer height of 0.20 mm and 180 mm/s printing speed (Ender 3 V3 SE, Creality, Shenzhen, China). The shells were assembled in modular fashion, featuring a separate skull base, anterior calvaria and posterior calvaria (Fig. 1a). The anterior calvaria was printed at 100% infill to facilitate drilling. The skull base and posterior calvaria could be recycled after each procedure.

### Brain Manufacturing

Using simplified 3-D models of the brain, molds that could be used for casting the left and right cerebral hemispheres were designed and 3-D printed in PLA (Fig. 1b). The molds were resized to ensure the hemispheres would fit precisely within the previously manufactured shell. Each mold featured a model of the frontal horn of the ipsilateral ventricle in its original anatomical location. This model could be removed after casting to create a hollow ventricular cavity. Casting was conducted using a 1.0% agarose polysaccharide gel with mechanical properties similar to brain tissue, based on a variation of a previously described method [22]. A high concentration of 1.0% agarose was selected for its stable structure over extended periods. For this, agarose powder was dissolved in distilled water at a 1.0% mass proportion and heated to 95 °C. After all the powder had dissolved, it was cooled to 50 °C and poured into the brain molds. After 3 h, the solution formed a firm gel that could be removed from the mold. The 3-D model of the frontal horn could then be gently removed to form the ventricular cavity. The hemispheres were then placed within the shell, which was closed using cyanoacrylate.

### Skin Manufacturing

Using cropped versions of the 3-D models of the skin, which contained the anterior half of the scalp and the upper facial region, molds were designed and 3-D printed to facilitate casting a skin layer (Fig. 1c). To cast the skin, water, glycerin and gelatin powder were mixed together in a 2:2:1 mass ratio. The mixture was heated to 80 °C and stirred until a homogenous solution formed. The solution was then cooled to 60 °C and poured in the skin molds. After 3 h, the skin sheet could be removed from the molds, draped over the facial region of the shell, and fixated using cyanoacrylate.

### Phantom Features

**Fig. 2:**
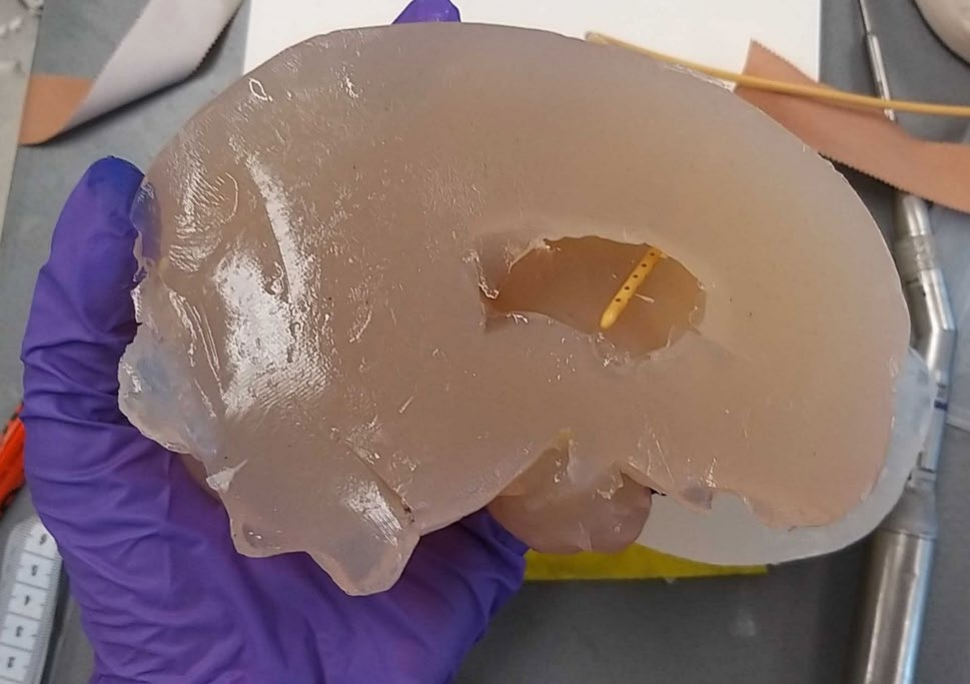
Visual inspection of EVD location within brain hemisphere

The resulting phantom formed a semi-realistic simulation model that could be used to conduct a bilateral EVD placement (Fig. 1d). The skin facilitated draping, marking, incision and retraction, while the skull could be used to drill a burr hole using either an automatic trephine or hand-cranked cranial access kit. An EVD could be inserted into the brain, subcutaneously tunneled and secured to the skin using staples or stitches. After conclusion of the procedure, the brain hemispheres could be removed to evaluate placement accuracy (Fig. 2). Alternatively, placement accuracy could be evaluated using Computed Tomography (CT) imaging. After disassembling the phantom, the posterior calvaria and skull base could be reused. Additionally, the brain and skin materials could be melted down and re-cast for repeated use. After an initial equipment investment of $288.28, the cost per phantom is $30.01 when materials are purchased in bulk (Table 1). 3-D models of the outer shell, brain mold and skin mold are provided in Electronic Supplementary Material 1.

**Table 1:** Cost overview of manufacturing a single phantom. Cost was converted to USD using an exchange rate of 1.08:1 USD:EURO (09/07/2024)

Expense	Retail quantity	Price per retail unit ($)	Quantity per phantom	Price per phantom ($)
Hot plate	1 unit	32.97	N/A	N/A
Stirring rod	8 pcs	8.99	N/A	N/A
2 L beaker	2 units	39.50	N/A	N/A
3-D printer (Ender 3 V3 SE)	1 unit	182.52	N/A	N/A
PLA (molds)	1.1 kg	24.30	N/A	N/A
PLA (phantom)	1.1 kg	24.30	350 g	7.73
Agarose powder	500 g	229.95	20 g	9.20
Glycerol	5 L	37.80	200 mL	1.51
Gelatin powder	250 g	15.44	100 g	6.18
4 mm Steel spheres	100 pcs	11.51	2 pcs	0.23
Pigments	17 ml	2.70	2 ml	0.32
Tape 50 mm	50 m	9.67	25 m	4.84
			**Equipment cost**	288.28
			**Per-phantom cost**	30.01

### Proof-of-Concept Experiment

**Fig. 3:**
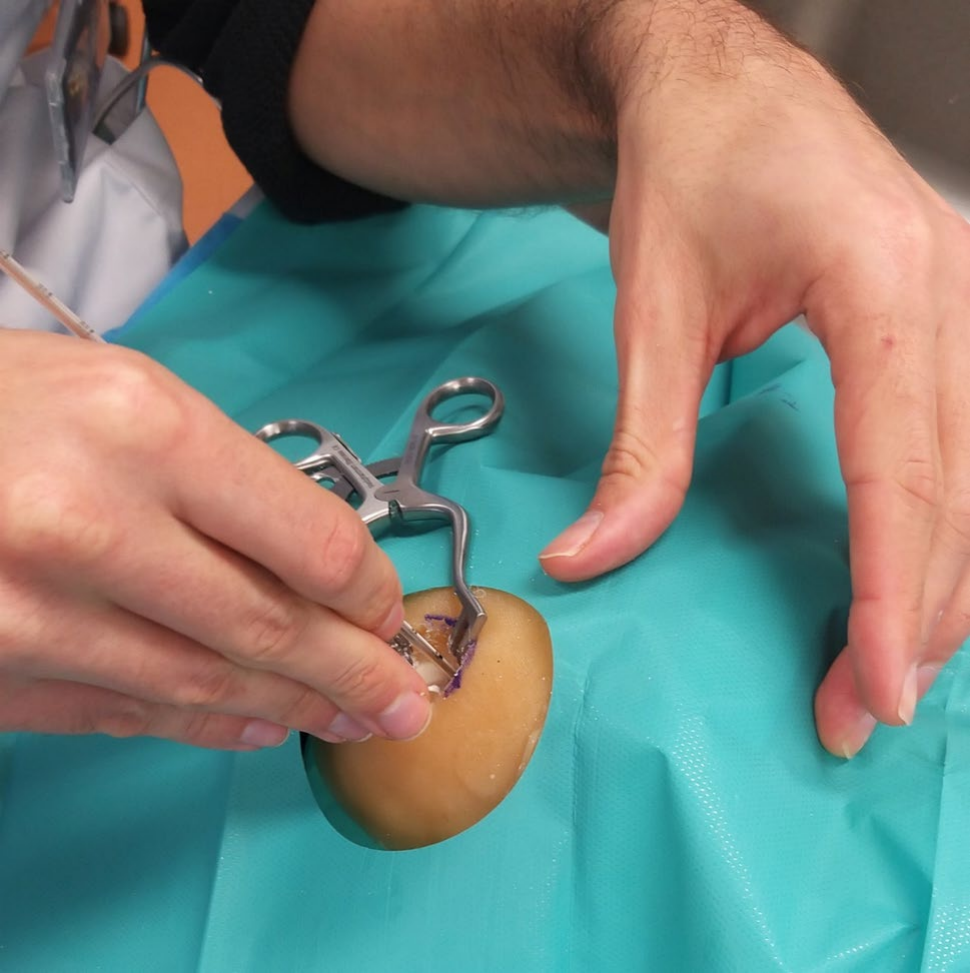
EVD placement on finished phantom

To assess the practical experience of performing EVD placement on the phantoms, four neurosurgeons, nine neurosurgical residents and two physician assistants each inserted bilateral EVDs in one of the two previously designed anatomical configurations, resulting in a total of 30 conducted EVD procedures. Placement was performed using the freehand technique at Kocher's point (Fig. 3). For this, Kocher’s point was bilaterally marked 10–11 cm posterior from the nasion and 3 cm lateral from the midline. Next, the phantom was draped using a fenestrated sterile sheet. Then, a semicircular incision was made using a no. 14 scalpel. A 14 mm burr hole was drilled using an electrical drill (Midas Rex, Medtronic, Dublin, Ireland). The EVD was inserted up until a depth of 65 mm relative to the skin and fixated using staples. Additionally, as a small-scale test, three individuals performed tunneling of the drain and closed the incision site using sutures. The proportion of optimally placed drains according to the Kakarla grading scale [26] was assessed using post-operative CT scans (Spectral CT, Philips, Amsterdam, The Netherlands). CT scans were conducted using helical acquisition with a slice thickness of 1 mm, slice spacing of 1 mm, tube current of 177 mA and tube voltage of 120 kVp.

### User Experience Questionnaires

To evaluate the user experience, all participants completed a User Experience (UX) questionnaire based on a survey previously described for assessing phantom quality in simulating EVD placement [14] (Electronic Supplementary Material 2). The questionnaire comprised 12 questions, with responses recorded on a 5-point Likert scale, covering evaluations of physical attributes, realism of the experience and its utility as a training tool. The assessment concluded with a global rating and general comments.

### Insertion Force Setup

**Fig. 4:**
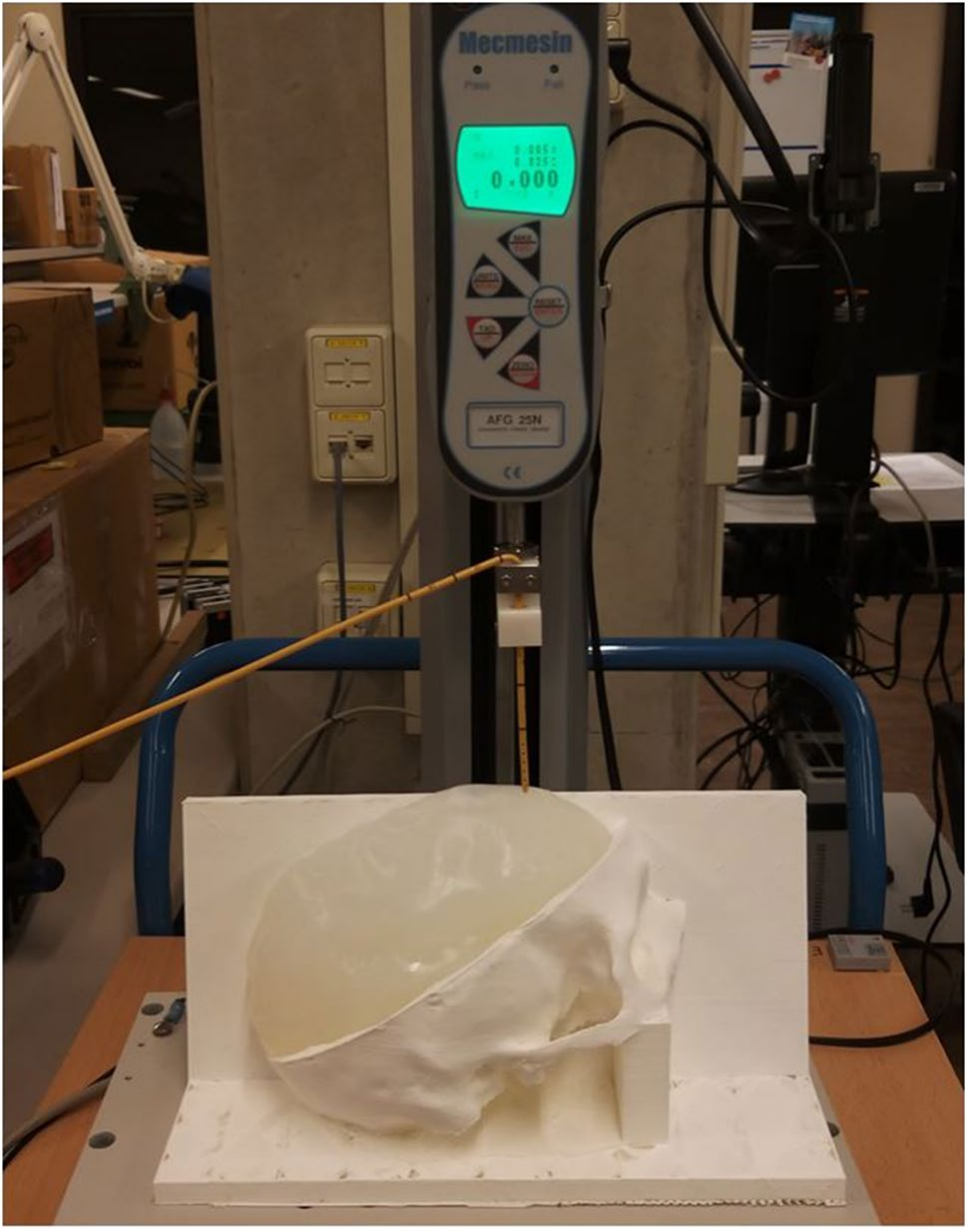
Mechanical setup for insertion force measurement

An experimental setup, based on a previously described system for measuring insertion force [27, 28], was developed to model the force profile during EVD penetration and insertion. The mechanical setup was used on a modified version of the phantom with varying levels of agarose concentration. The setup consisted of a linear force tester (MultiTest 2.5-dV, Mecmesin, Slinfold, United Kingdom) integrated with a 25 N force gauge (AFG 25, Mecmesin, Slinfold, United Kingdom) with a 0.005 N resolution and ± 0.025 N accuracy. The linear force tester was used to insert a 3 mm EVD (Codman, Integra LifeSciences, Princeton, USA) with an internal stiff stylet at a perpendicular angle into the agarose brain at the approximate position of Kocher’s point (Fig. 4). The catheter was fixated to the force gauge at the 10 cm mark, which is the estimated position of a surgeon’s hand. Insertion occurred at a constant velocity of 0.3 mm/s up to a depth of 45 mm, while the force opposing the EVD was measured at a rate of 80 samples per second. Trials were conducted at a temperature of 22 °C for phantoms with agarose concentrations of 0.6% and 1.0%.

## Results

### Proof-of-Concept Results

**Fig. 5:**
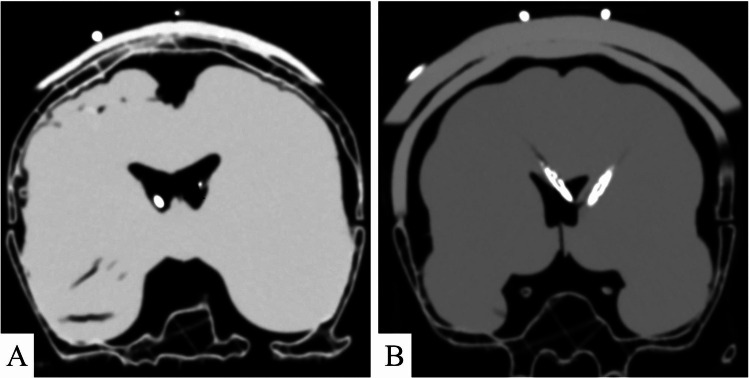
A Example of bilateral Kakarla 1 placement. B Example of Kakarla 2 placement (right hemisphere) and Kakarla 3 placement (left hemisphere)

Kocher’s point planning using anatomical landmarks was carried out for all 30 procedures without functional problems, except slight diffusion of the surgical marker’s ink. Incision and retraction was successfully performed for all procedures, although retraction caused tearing of the skin in some cases. Drilling was conducted for all procedures without significant problems, except slight interference of molten PLA within the surgical site. EVD placement was completed for all 30 procedures, with 23 procedures (76.7%) exhibiting optimal placement with a Kakarla score of 1, 1 procedure (3.3%) exhibiting a Kakarla score of 2 and 6 procedures (20.0%) exhibiting a Kakarla score of 3 (Fig. 5). Tunneling and closing was performed in 3 procedures without functional problems.

### User Experience Results

**Fig. 6:**
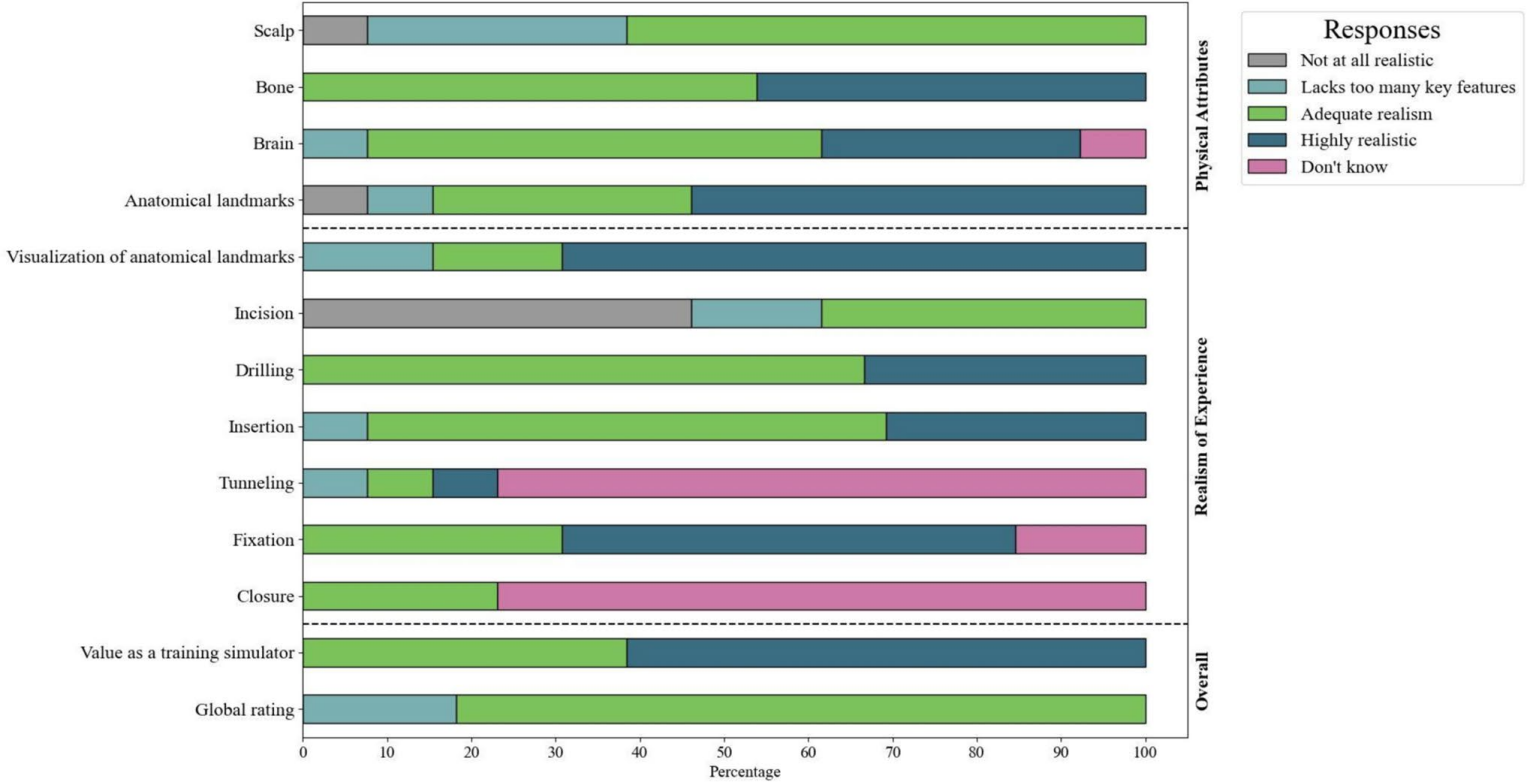
Average responses per questionnaire item

Thirteen participants completed the UX questionnaire (Fig. 6). On a scale from 1 to 4, the respondents provided average ratings of 3.1 for the ‘physical attributes’ category, 3.3 for the ‘realism of experience’ category, 3.6 for ‘value as a training tool’ and 2.8 for overall satisfaction. The highest average score for an individual item was 3.63 for 'Realism of EVD fixation', while the lowest was 1.91 for 'Realism of incision and retraction of skin'. Comments generally praised the added value of practicing spatial awareness during EVD placement based on external anatomical landmarks. However, participants criticized the thickness and tearing of the skin layer during incision and retraction.

### Insertion Force Modelling

**Fig. 7:**
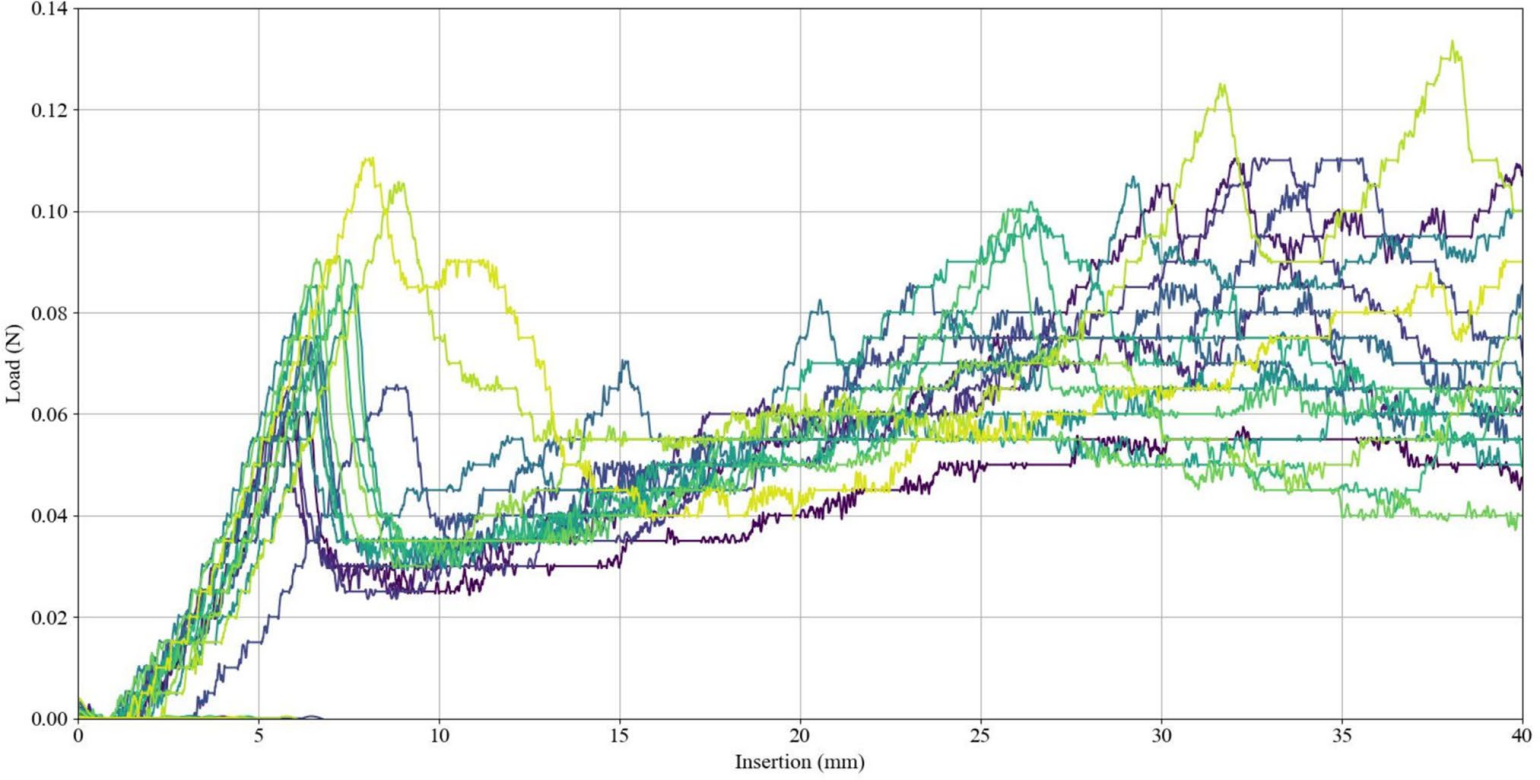
Insertion forces exerted on 0.6% agarose during catheter insertion

**Fig. 8:**
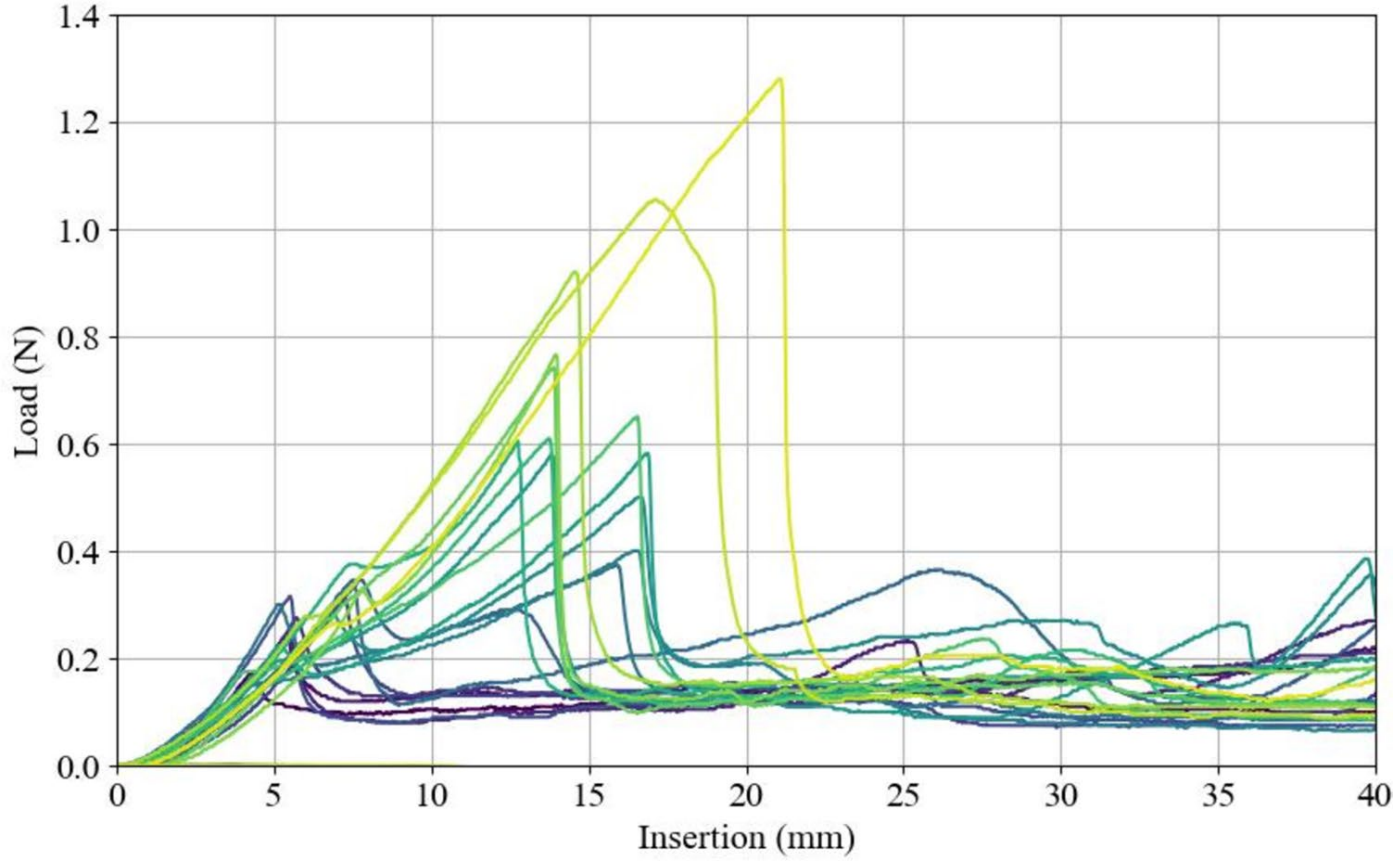
Insertion forces exerted on 1.0% agarose during catheter insertion

Eighteen runs of force measurement during catheter insertion were completed for the 0.6% agarose sample. Fifteen runs were completed for the 1.0% agarose sample. For the insertion force profile, all trials showed an initial force transient during penetration of the cortical surface, followed by a stable phase with varying fluctuations of force due to drag forces. The median penetration peak force for 0.6% agarose was 0.083 N (IQR = 0.019), followed by a median stable phase of 0.060 N (IQR = 0.020) with a continuous increase in load due to drag forces (Fig. 7). The median penetration peak force for 1.0% agarose was 0.50 N (IQR = 0.34), followed by a stable phase with a median force of 0.14 N (IQR 0.06 N) with considerable variation in force fluctuations depending on the insertion angle and entry point (Fig. 8).

## Discussion

In this study, we outline the development of a resource-efficient patient-specific phantom for simulating EVD placement. Validation by neurosurgical residents revealed a suboptimal placement rate of 23.3%, mirroring rates observed in clinical settings [3, 29]. Feedback from neurosurgeons and neurosurgical residents was generally favorable, particularly noting the realism of the burr hole drilling, EVD insertion and EVD fixation. However, the realism of incision and retraction of the skin layer was rated low.

The insertion force profile of 0.6% agarose was consistent with previous literature, which reported a mean penetration force of 0.085 N and a drag force increasing between 0.04 and 0.09 N[22]. This profile is also similar to forces exerted on a 2.5 mm probe passing through real brain tissue, which has been reported to exhibit a penetration force of 0.010 to 0.015 N and stable drag force of approximately 0.08 N [28]. This similarity supports the notion of realistic tactile feedback for this concentration. However, while we established that 0.6% agarose has tactile properties similar to real brain tissue, this concentration tends to shrink over time, making a 1.0% agarose solution more suitable for scenarios involving long intervals between usage.

### Advantages of Model

Our phantom provides realistic tactile properties and anatomical landmarks, resulting in an authentic simulation experience. Additionally, while this study utilized post-operative imaging in this study to determine the exact position of the EVD, it is also possible to remove the brain hemispheres for direct visual inspection of the drain location. This capability eliminates the dependence on imaging and enables rapid assessment of placement accuracy.

The manufacturing efficiency, cost-effectiveness and adaptability to different clinical scenarios make the phantom more suitable for settings that require high-volume creation tailored to a wide variability of clinical cases without requiring significant technical expertise, which is of particular value in low-resource settings or scientific research. In a research setting, the phantom could be used to validate and compare new surgical devices for EVD procedures. The straightforward design methods using real imaging data enable the phantom to be rapidly adapted to simulate varying technical difficulties of EVD placement based on the patient’s anatomy, such as the size and shape of the ventricles. With minor modifications, the phantom could be adapted to facilitate the simulation of other trajectory-based procedures, such as deep brain stimulation or brain biopsies.

### Literature

Phantoms designed for simulating EVD placement or third ventriculostomy have been previously described in literature [15–21]. Electronic Supplementary Material 3 highlights the differences between our phantom and these existing models. While many of the previously described phantoms provide anatomically realistic simulations, they often demand greater technical expertise and more man-hours for production compared to our method [15, 17–21]. Furthermore, their production costs tend to be higher due to more expensive materials or manufacturing techniques [14, 15, 17]. Lastly, previously described phantoms either lack a skin layer entirely [15, 17] or feature a skin layer on the calvaria only [14, 16], which limits the use of anatomical landmarks during EVD placement.

Compared to certain phantoms described in literature, our phantoms have reduced structural anatomical detail, such as the omission of the dura mater, choroid plexus and cerebrospinal fluid [15, 16, 18–21]. We believe that highly-demanding simulations conducted in low quantities (such as neuro-endoscopic surgery) might benefit more from realistic, intricate models with reusable components. Furthermore, questionnaire responses and experiences during the experiments suggest that the realism of the skin layer requires improvement. Additionally, we observed during early testing that the coronal suture is not palpable through the skin and cannot be used as an anatomical landmark. Thus, reducing the skin layer thickness and preventing the skin tearing that occurs during retraction are needed.

### Future Perspectives

In future iterations, we plan to enhance the phantom's realism in simulating EVD placement. To increase simulation fidelity, we aim to incorporate a watertight ependymal lining within the ventricles, featuring a lightly pressurized liquid to authentically simulate the tactile sensation experienced during ventricular puncture and subsequent return of CSF. Additionally, we are investigating the development of a skin layer that envelops the entire skull, including the ears, to enable utilizing auricular landmarks. We will focus on employing material composites with greater shear resistance to reduce tearing. Moreover, we intend to optimize the manufacturing process by employing a unified mold for both cerebral hemispheres and utilizing mold materials capable of withstanding higher temperatures, facilitating faster casting. Furthermore, we aim to improve the ease of establishing the drain position by integrating a stereotactic measuring frame or making the phantom compatible with electromagnetic tracking. We also aim to explore the design of phantoms with greater anatomical variety, specifically for challenging EVD procedures such as those involving midline shift or slit-like ventricles. To enhance the validation of the phantom through proof-of-concept experiments, we aim to establish a learning curve for varying levels of expertise by having each participant place multiple EVDs in a randomized order of difficulty and left/right placement.

## Conclusion

In conclusion, our phantom offers a cost-effective and low-complexity solution for simulating EVD placement. The model is particularly advantageous for high-volume applications, such as scientific research or training programs. Additionally, it serves as a versatile template allowing investigators to customize phantoms based on specific imaging data or clinical applications.

## Supplementary Information

Below is the link to the electronic supplementary material.

Supplementary file1 stl meshes of outer shell, brain mold and skin mold of phantom (ZIP 114975 KB)

Supplementary file2 Questionnaire for evaluating User Experience of EVD simulation using our phantom (DOCX 5672 KB)

Supplementary file3 Comparison of characteristics between our phantom and other previously described EVD simulation phantoms (XLSX 11 KB)

## Data Availability

No datasets were generated or analysed during the current study.
