# Supplementary material for: Development and Validation of a Neurosurgical Phantom for Simulating External Ventricular Drain Placement
Source: J Med Syst. 2025 Jan 3;49(1):1. doi: 10.1007/s10916-024-02133-4 (PMC11698783; doi:10.1007/s10916-024-02133-4)
Supplement: Supplementary file 2 — Supplementary file2 Questionnaire for evaluating User Experience of EVD simulation using our phantom (DOCX 5672 KB) [file 10916_2024_2133_MOESM2_ESM.docx]

*Validation of a low cost patient-specific EVD simulation phantom*

**Phantom Study - Phantom Evaluation**

**Please rate the following aspects of placing an External Ventricular Drain (EVD) within the phantom using the freehand technique:**

Scalp

Don't know

Very relevant (4)

Some relevance (3)

No relevance (1)

Little relevance (2)

Not at all realistic (1)

Value of simulator as a training tool

Scalp closure

Fixation of EVD

Incision and retraction of skin

Identifying landmarks

Visualization of landmarks

Brain tissue texture

Bone

Highly realistic, no changes needed (4)

Lacks too many key features to be useful (2)

Adequate realism, but could be improved (3)

Don't know

PHYSICAL ASPECTS

Drilling of bone

Inserting EVD

REALISM OF EXPERIENCE

OVERALL EXPERIENCE

**Global—Please check the one statement below with which you most agree:**

This simulator requires extensive improvements before it can be considered for use in ventriculostomy placement training.

This simulator requires minor adjustments before it can be considered for use in ventriculostomy placement training.

This simulator can be used in training ventriculostomy placement as is, but could be improved slightly. Testtesttes

This simulator can be used in ventriculostomy placement training with no improvements made.

**General comments: why is this simulator useful / not useful for ventriculostomy training?**
